# Fertile Hybrids Could Aid Coral Adaptation

**DOI:** 10.1002/ece3.70570

**Published:** 2024-11-20

**Authors:** Annika M. Lamb, Lesa M. Peplow, Wing Yan Chan, Zoe J. Crane, Glenn A. Everson, Peter L. Harrison, Talley E. Hite, Ary A. Hoffmann, Craig A. Humphrey, Lonidas P. Koukoumaftsis, Madeleine J. H. van Oppen

**Affiliations:** ^1^ Australian Institute of Marine Science Cape Cleveland Queensland Australia; ^2^ School of Biosciences University of Melbourne—Biosciences 4, The University of Melbourne Parkville Victoria Australia; ^3^ AIMS@JCU James Cook University Townsville Queensland Australia; ^4^ Marine Ecology Research Centre Southern Cross University Lismore New South Wales Australia; ^5^ School of Biosciences, Bio21 Institute University of Melbourne Melbourne Victoria Australia

**Keywords:** coral, coral reef, evolution, hybrid

## Abstract

Fertile hybrids can enhance the adaptive capacity and resilience of species under stress by increasing genetic diversity within populations, masking the effects of deleterious recessive alleles, and facilitating the introgression of beneficial genetic variants into parental species. However, many hybrids are infertile. We compared the fertility of aquarium‐reared F1 hybrid and purebred corals of the species *Acropora loripes* and *Acropora kenti* and examined the viability of early life stages of second‐generation (F2) hybrid and back‐crossed planula larvae and recruits. The F1 hybrids spawned viable gametes and the F2 hybrid and back‐crossed embryos developed into planula larvae and settled to become sessile coral recruits. The F1 hybrids had greater reproductive fitness than the F1 *A*. *loripes* purebred stock in an aquarium environment based on their probability of spawning and their fertilization success in crosses using their gametes. Interspecific coral hybrids can therefore be fertile and have high reproductive fitness, which could benefit the persistence of threatened coral reefs.

## Graphical abstract

Fertile hybrids have the potential to enhance the resilience and adaptative potential of populations. We show that interspecific coral hybrids can be fertile and thus may support the persistence of coral reefs into the future.

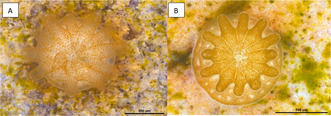

## 1 Introduction

Stressors such as rising sea surface temperatures, ocean acidification, and pollution are driving declines in coral populations across the globe (De'ath et al. 2012; Doney et al. 2009; Hoegh‐Guldberg 1999; Souter et al. 2021). Declines in populations of reef‐building corals threaten the immense biological diversity of coral reefs (Reaka‐Kudla 1997) and the billions of dollars' worth of ecosystem services they provide (Spalding et al. 2017; van Zanten, van Beukering, and Wagtendonk 2014; Eddy et al. 2021). For coral reefs to persist into the future, corals must adapt to extreme environmental change.

Interspecific hybridization involves the interbreeding of individuals from different species to generate offspring and has the capacity to facilitate adaptation (Chan, Hoffmann, and van Oppen 2019; VanWynen et al. 2021). Interspecific hybrids inherit alleles from two different species that in combination can increase their fitness relative to that of the purebred parental species, a phenomenon termed hybrid vigor, adding to the genetic diversity of populations (Baskett and Gomulkiewicz 2011; Willis et al. 2006; Kitchen et al. 2019). Hybrids also allow the exchange of beneficial alleles between their parental purebred species through backcrossing if they are sexually viable (Baskett and Gomulkiewicz 2011; Hamilton and Miller 2016). The beneficial impact of the transfer of genetic information between species via backcrossing has been demonstrated across biological systems (Steensels, Gallone, and Verstrepen 2021; Hubner et al. 2019; Huerta‐Sánchez et al. 2014).

The contribution of interspecific hybrids to species adaptation is partially dependent on their reproductive capacity, and many hybrids are infertile (mules and hinnies are a classic case of this; Taylor and Short 1973). The chromosomal compatibility of the two parental purebred species will impact the fertility of first‐generation (F1) hybrids. If individuals inherit different numbers of chromosomes from their mother and father, errors can occur in meiosis during the formation of gametes (Benirschke, Brownhill, and Beath 1962). Further, individuals who are heterozygous for chromosomal rearrangements such as tandem fusions, inversions, and translocations can produce gametes that have genetic duplications and deficiencies due to erroneous recombination (White 1977). Such gametes may be inviable or produce second‐generation (F2) offspring with reduced fitness (Rieseberg 2001). The reproductive capacity of F1 hybrids must therefore be tested through observations of their gamete development, experimentally crossing hybrids to generate an F2 generation, and if successful cross‐fertilization occurs, assessing the viability of this generation.

Corals can hybridize naturally (Fogarty 2012; Richards et al. 2008; van Oppen et al. 2002) and hybrid vigor has been observed in coral hybrids in some environments (VanWynen et al. 2021; Fogarty 2012; Chan et al. 2018; Willis et al. 1997). In vitro fertilization of gametes from *Acropora loripes* with *Acropora kenti* or *Acropora florida* with *Acropora sarmentosa* from the Great Barrier Reef (GBR) demonstrated high rates of fertilization between these pairs of species (Chan et al. 2018). While the latter pair may hybridize in nature, disparate spawning times are expected to naturally restrict interbreeding between *A*. *loripes* with *A*. *kenti*. The hybrids produced through crossing these pairs grew and survived as well or better than their purebred counterparts under ambient and elevated temperatures and *p*CO2 levels in a laboratory environment (Chan et al. 2018). Interspecific hybridisation may be a novel option for managers to produce genetically diverse and resilient coral stock for coral reef restoration initiatives.

The introgression of genetic variants amongst coral lineages is indicative that some coral hybrids are fertile (van Oppen et al. 2002). However, quantitatively assessing the reproductive viability of coral hybrids requires experimentally crossing corals to produce an F1 generation, growing the F1 to reproductive maturity and (back‐) crossing the F1 generation to produce F2 and backcross generations; this feat has rarely been achieved (Craggs et al. 2020). Isomura, et al. (2016) reared two *Acropora florida* × *A*. *intermedia* F1 hybrid colonies for seven years until they reached sexual maturity and demonstrated that their gametes were capable of crossing with one another and backcrossing with colonies of their parental species to produce F2 and backcross planula larvae. Here, we describe the spawning behavior of the F1 *Acropora loripes* × *A*. *kenti* hybrids produced by Chan, et al. (2018), successfully cross their gametes, and demonstrate the viability of the early life stages of an F2 hybrid and a backcrossed generation.

## 2 Results

In 2015, *Acropora loripes* eggs were crossed with *A*. *loripes* sperm to generate *A*. *loripes* purebred offspring (LL_F1_), and *A*. *loripes* eggs were mixed with *A*. *kenti* sperm to generate LK_F1_ hybrid offspring (Chan et al. 2018). These corals were grown in the National Sea Simulator (SeaSim) at the Australian Institute of Marine Science (AIMS) for the entirety of their lives and became the F1 parental subjects of this experiment. The corals were studied prior to, during, and after the predicted annual spawning periods for *A*. *loripes* and *A*. *kenti* on the GBR between 2019 and 2021. Gametogenesis, spawning activity, and gamete viability of the LL_F1_ and LK_F1_ colonies were compared, and the gametes of the F1 colonies were crossed to assess their fertility and the viability of early life stages of an F2 generation.

### 2.1 F1 Gametogenesis

**FIGURE 1:**
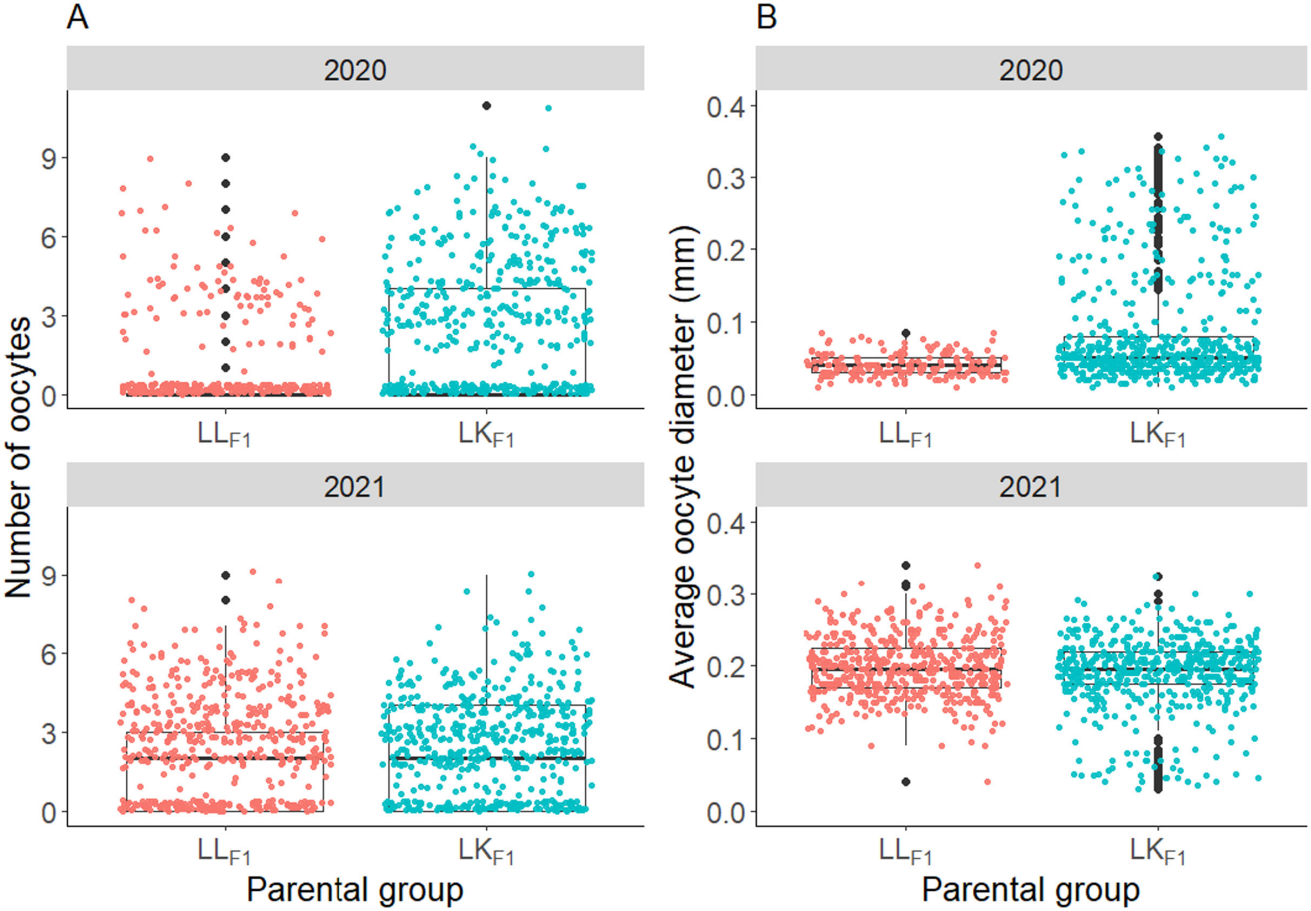
Box plots depicting the distribution of (A) the number of oocytes in the mesenteries of LL_F1_ purebred (orange) and LK_F1_ hybrid (blue) colonies, where each dot represents the oocyte count in a single mesentery, and (B) the mean diameter of oocytes in the LL_F1_ purebred and LK_F1_ hybrid colonies, where each dot represents the average diameter (mm) of a single oocyte. The horizontal lines of the boxes represent the lower quartile, median, and upper quartile values, the “whiskers” represent the extreme values, and dots represent single outlier datapoints.

Polyp dissections showed that the LL_F1_ and LK_F1_ colonies contained oocytes, demonstrating that the F1 parental groups were capable of gametogenesis in 2020 and 2021. Sterile zones that typically occur in the branch tips of acroporids were not observed in the dissected fragments (Randall, Giuliano, and Page 2021; Wallace 1985). A zero‐inflated generalized linear mixed effects model was used to compare the number of oocytes produced by the LL_F1_ and LK_F1_ corals in 2020 and 2021. The mesenteries of the colonies with eggs contained 1.4 times the number of eggs in 2020 that they did in 2021 (*Z* = −3.574, *p* < 0.001; Figure 1A). However, a mesentery in a colony was 14.1 times more likely to contain eggs in 2021 than in 2020 (*Z* = −10.374, *p* < 0.001; Figure 1A). There was no significant difference in the number of oocytes per mesentery between the LK_F1_ and LL_F1_ colonies in 2020 (*Z* = −1.026, *p* = 0.305) or 2021 (*Z* = −0.413, *p* = 0.679), although, a mesentery of a LK_F1_ colony was 4.9 times as likely to contain eggs as a mesentery of a LL_F1_ colony (*Z* = −9.166, *p* < 0.001; Figure 1A). Furthermore, the interaction between the parental group (LK_F1_ or LL_F1_) and year of sampling (2020 or 2021) had a significant effect on the number of eggs in the mesentery of a colony (*Z*‐score = −2.718, *p* = 0.007). The difference between the number of eggs per mesentery of the LK_F1_ (median = 2, range 0–11) and LL_F1_ colonies (median = 0, range 0–9) was larger in 2020 than the difference between the number of eggs per mesentery of the LK_F1_ (median = 0, range 0–9) and LL_F1_ colonies (median = 0, range 0–9) in 2021. The effect of parental group on the odds of a mesentery containing eggs was greater in 2021 than in 2020 (*Z*‐score = 3.308, *p* < 0.001).

Linear mixed effects models (LMMs) were used to compare the size of the oocytes in the LL_F1_ and LK_F1_ corals. In 2020, the LL_F1_ oocytes (median = 0.04 mm, range = 0.01–0.09 mm) were smaller in average diameters than the LK_F1_ oocytes (median = 0.05 mm, range = 0.01–0.36 mm; *t*(36.4) = −4.649, *p* < 0.001; Figure 1B). There was no significant difference in the average diameter of the LL_F1_ (median = 0.20 mm, range = 0.04–0.34 mm) compared with the LK_F1_ (median = 0.20 mm, range = 0.03–0.42 mm) oocytes in 2021 (*t*(31.4) = 1.611, *p* = 0.387; Figure 1B). The average diameters of the oocytes in samples taken 3 weeks prior to the full moon in November 2020 were smaller than the average diameters of the oocytes in samples taken 9 days prior to the full moon in December 2021 for the LL_F1_ (*t*(759.8) = −23.244, *p* < 0.001) and LK_F1_ (*t*(1146.1) = −11.078, *p* < 0.001; Figure 1) corals.

### 2.2 F1 Spawning Activity

On 18/12/2019 (6 days following the full moon), one of the 4‐year‐old LK_F1_ corals spawned and constituted the first and only spawning observation in the LL_F1_ and LK_F1_ corals prior to 2021. Details of the 2019 spawning observations are outlined in Appendix SI.

**FIGURE 2:**
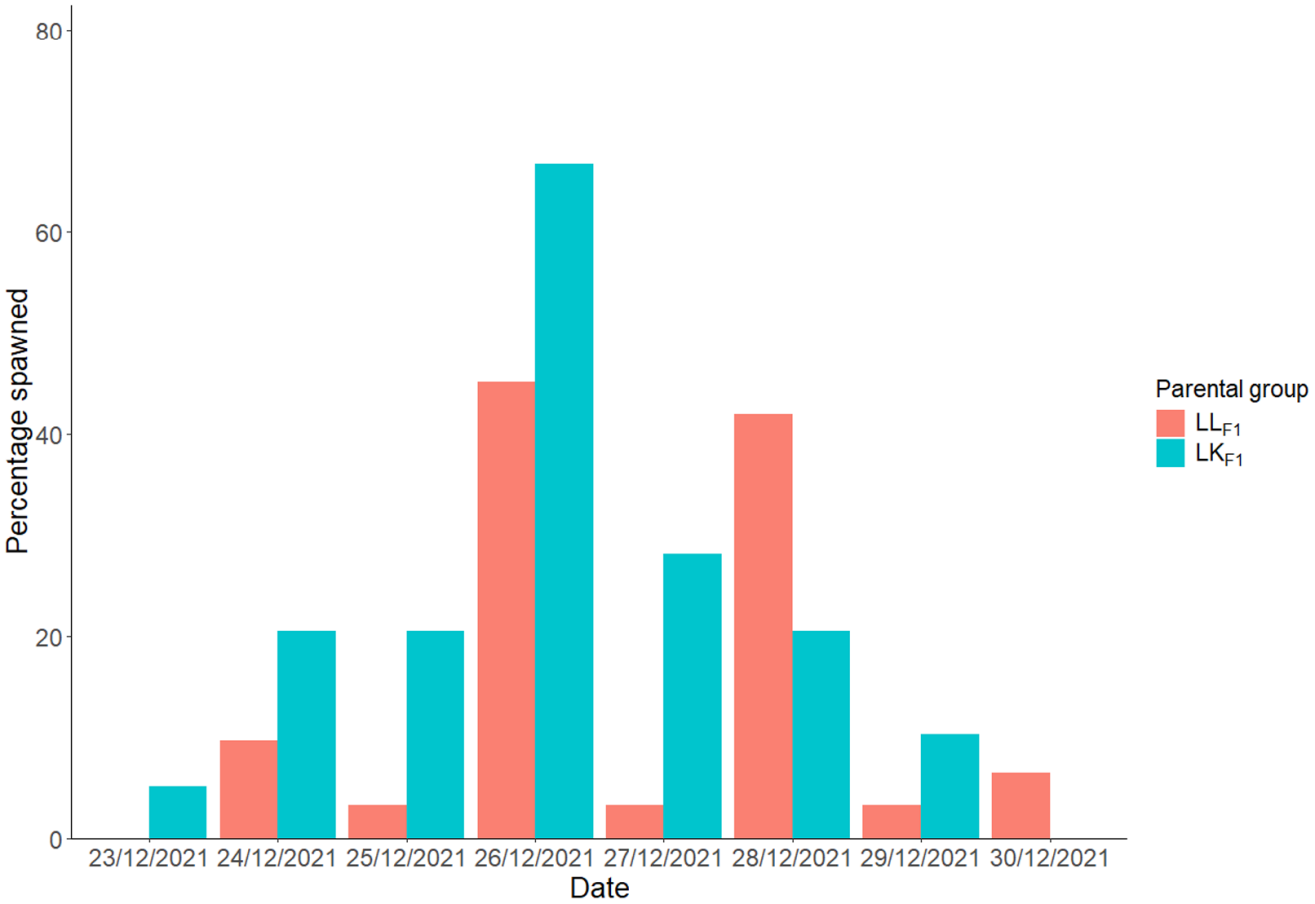
Bar chart of the percentage of the 39 LK_F1_ hybrid and 31 LL_F1_ purebred colonies spawning on each night of the December 2021 spawning period.

**FIGURE 3:**
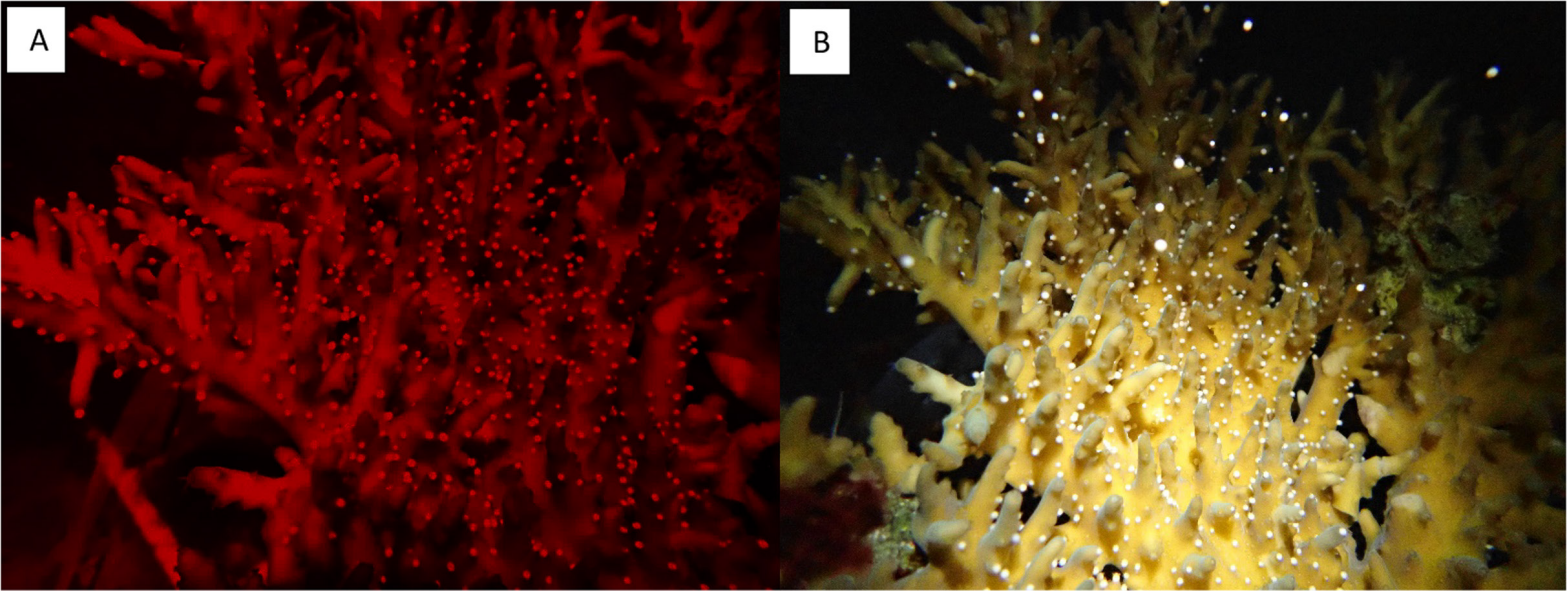
Photographs of a LK_F1_ colony (A) setting and (B) spawning in December 2021, taken with an Olympus TG‐5 camera. Note that the image of the coral setting was taken under red light to minimize disruption of spawning behavior.

Between 23/12/2021 and 30/12/2021 (4–11 days following the full moon on the 19/12/2021), 21 of the 31 LL_F1_ corals and 32 of the 39 LK_F1_ corals growing in the SeaSim spawned (Table S1). A LMM was used to compare the spawning times of the LL_F1_ and LK_F1_ in 2021; the LK_F1_ hybrids began spawning at the same time as the LL_F2_ purebred colonies (*T* = 0.76, *p* = 0.452), between 137 and 166 min after sunset. A GLMM was used to compare the number of LL_F1_ and LK_F1_ that spawned over the eight nights of the 2021 spawning; more of the LK_F1_ hybrids spawned than the LL_F1_ purebred colonies (*Z* = 2.144, *p* = 0.032; Figure 2) such that on a given night, a LK_F1_ hybrid was 2.0 times as likely to spawn as a LL_F1_ purebred. The LK_F1_ hybrid coral setting and spawning behavior was characteristic of the maternal species (Figure 3).

### 2.3 F2 Fertilization Success and Viability

The eggs released by the colonies in 2021 were pigmented pink, and both the eggs and sperm of all LL_F1_ and LK_F1_ colonies tested could be cross‐fertilized. Fertilization was not observed in any egg samples without sperm, indicating that there was no sperm contamination of the eggs used in the fertilization tests. We also tested for self‐fertilization. In 2021, one of the eight tested LL_F1_ and one of the 10 tested LK_F1_ colonies self‐fertilized at a rate of 1%, and three LL_F1_ and two LK_F1_ colonies self‐fertilized at a rate of 100% in duplicate reactions. All other self‐fertilization attempts were unsuccessful.

**FIGURE 4:**
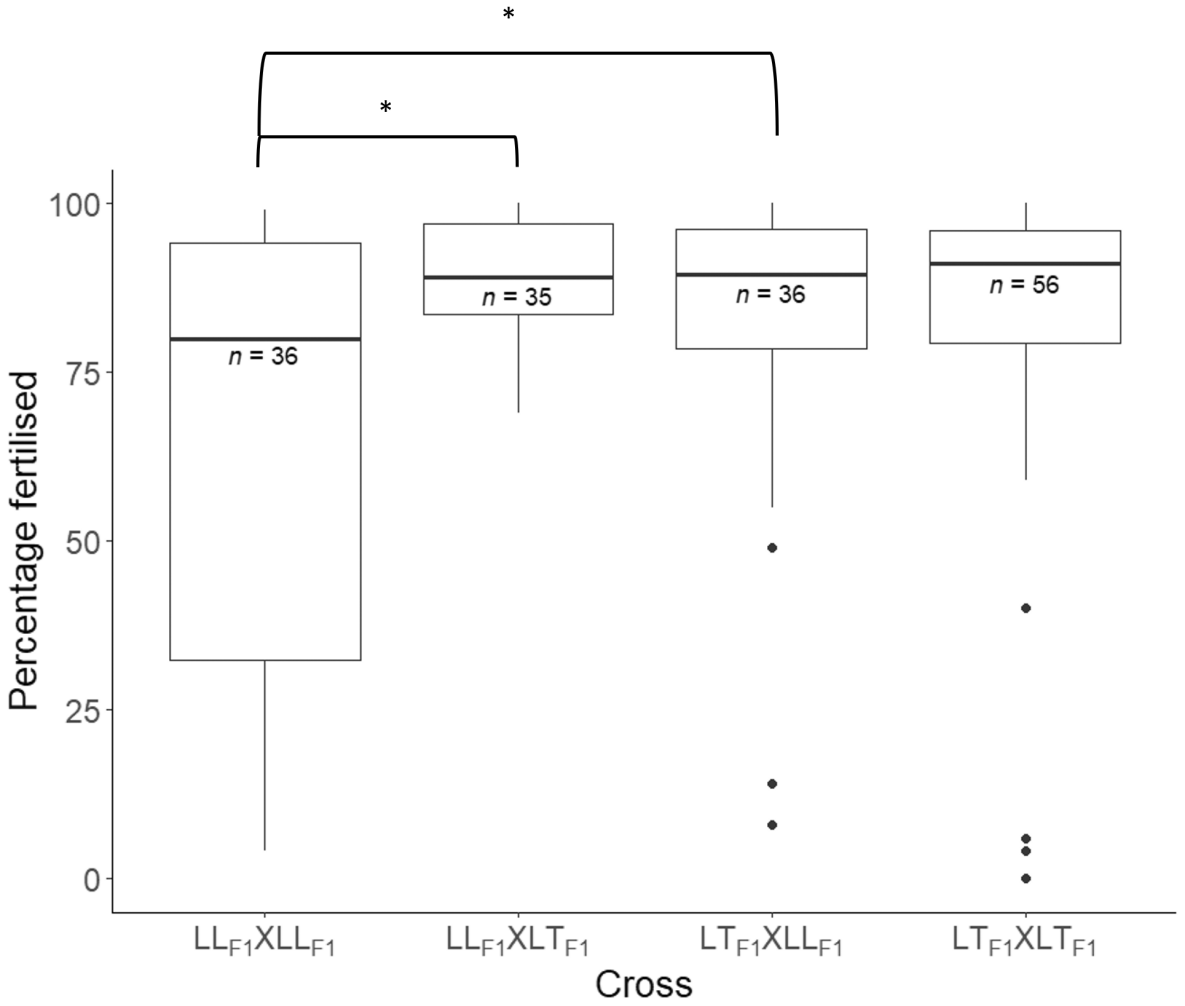
Box plots depicting the distribution of fertilization success (percentage of multicell embryos post fertilization) of the crosses conducted between LL_F1_ and LK_F1_ colonies in December 2021. The dam and sire F1 parental group are listed first and second, respectively, in the cross labels on the *x*‐axis. The horizontal lines of the boxes represent the lower quartile, median, and upper quartile values, the “whiskers” represent the extreme values, and dots represent single outlier datapoints. Sample sizes (number of fertilization reactions) are shown below each median line for each offspring group; note that duplicate counts were conducted for each unique pair of colonies crossed and that one duplicate was missing for the cross LL_F1_ × LK_F1_. Significant differences in fertilization success amongst the crosses have been inferred from Bayesian generalized linear mixed effects modeling and are annotated on the figure with *.

In December 2021, gravid wild *A*. *loripes* and *A*. *kenti* colonies were not available for crossing at AIMS, hence the LL_F1_ and LK_F1_ corals could only be crossed with one another to test the viability of their gametes and the ability of the LK_F1_ corals to cross with other LK_F1_ corals and backcross with purebred *A*. *loripes* to produce F2 offspring. The following crosses were conducted in duplicate: 18 crosses between unique combinations of the eggs and sperm of eight different LL_F1_ purebreds (LL_F1_ × LL_F1_) to produce LLLL_F2_ corals, 18 crosses between unique combinations of the eggs of one of six LL_F1_ purebred and the sperm of one of eight LK_F1_ hybrid colonies (LL_F1_ × LK_F1_) to produce LLLK_F2_ corals, 18 crosses between unique combinations of the eggs of one of eight LK_F1_ hybrid and the sperm of one of six LL_F1_ purebred colonies (LK_F1_ × LL_F1_) to produce LKLL_F2_ corals, and 28 crosses between unique combinations of the eggs and sperm of nine different LK_F1_ hybrids (LK_F1_ × LK_F1_) to produce LKLK_F2_ corals (Table 1). A Bayesian generalized linear mixed effects model (BGLMM) was used to test the difference in fertilization success amongst the four different groups of crosses. The R‐hat convergence diagnostics of the BGLMM were 1.00, the bulk effective samples sizes (2316–2488) and tail effective sample sizes (2238–2354) of the model estimates were large, the posterior distributions of the model estimates were unimodal and normally‐distributed, and the time‐series plots of the model estimates for each chain tracked with one another. The results of the BGLMM indicate that the LL_F1_ × LL_F1_ cross had significantly lower fertilization success than the LL_F1_ × LK_F1_ cross (highest posterior density interval (HPD) does not overlap with zero; HPD = −0.941 to −0.080; Figure 4) and LL_F1_ × LL_F1_ cross (HPD = −1.342 to −0.027; Figure 4). All other pairwise comparisons of fertilization success between the crosses were not statistically significantly different (HPDs overlapped with zero; Figure 4), indicating that the fertilization success of the LK_F1_ × LK_F1_ crosses were intermediate compared to those of the LL_F1_ × LL_F1_ and LL_F1_ × LK_F1_ and LK_F1_ × LL_F1_ crosses.

**TABLE 1:** Offspring groups resulting from the crosses of the eggs and sperm of the various parental coral groups conducted in the 2021 December spawning season. Number of crosses conducted amongst unique pairs of colonies are recorded (*N* of crosses).

Dam	Sire	Offspring group	*N* of crosses
LK_F1_	LL_F1_	LKLL_F2_	18
LL _F1_	LK_F1_	LLLK_F2_	18
LL _F1_	LL_F1_	LLLL_F2_	18
LK_F1_	LK_F1_	LKLK_F2_	28

**FIGURE 5:**
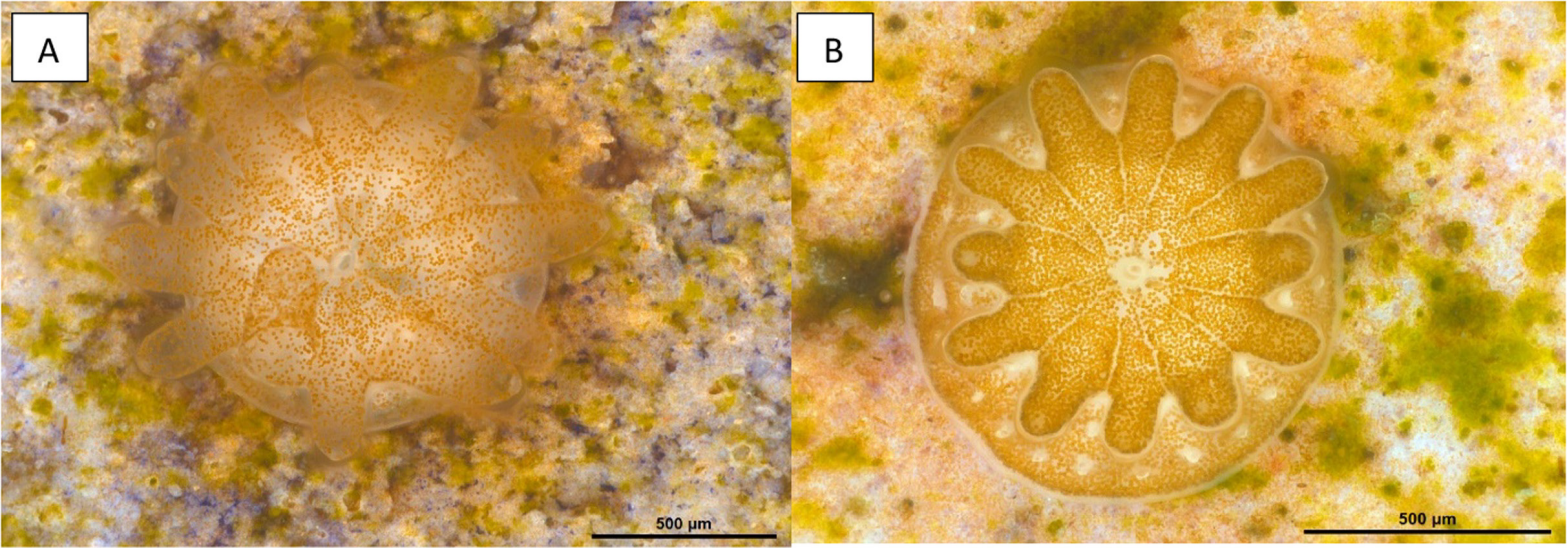
Images of F2 colonies taken using the Leica Stereo Microscope MZ16A that depict a LLLL_F2_ and LKLL_F2_ coral recruit, respectively, taken approximately 1 month after they settled in 2021; the Symbiodiniaceae photosymbionts can be seen as scattered, golden/brown dots in these images.

The corals reared from successful crosses conducted in 2021—LLLL_F2_, LLLK_F2_, LKLL_F2_ and LKLK_F2_—developed through to their planula larval stage, settled, became infected with Symbiodiniaceae photosymbionts, and survived in the SeaSim to the time of this publication (Figure 5).

## 3 Discussion

### 3.1 F1 *Acropora* Coral Hybrids Are Fertile

The successful spawning of the LK_F1_ hybrids and high fertilization success of their gametes demonstrates that interspecific *Acropora* coral hybrids can be fertile. This is in keeping with genetic data that suggests gene flow occurs amongst coral species within several genera including *Acropora* (van Oppen et al. 2000, 2001; Diekmann et al. 2001; Kitchen et al. 2020; Vollmer and Palumbi 2007). Microsatellite and single nucleotide polymorphism sequencing data have been used to identify F2 backcrossed corals that demonstrate the Caribbean F1 hybrid, *A*. *prolifera*, is capable of backcrossing with both of its parental purebred species (Kitchen et al. 2020; Japaud et al. 2019). Two F1 hybrids produced experimentally between *A*. *intermedia* and *A*. *florida* from Japan have also been shown to be fertile (Isomura et al. 2016). Thus, hybrid fertility is not restricted to the *A*. *loripes* × *A*. *kenti* hybrid studied here.

The spawning behavior of the LK_F1_ hybrids matched that expected from wild colonies of the parental species. The LK_F1_ and LL_F1_ colonies set and spawned in the manner characteristic of acroporids, despite having been reared in an aquarium. In 2021, the 32 spawning LK_F1_ hybrids began releasing gametes simultaneously with LL_F1_ colonies and at times characteristic of their maternal species, *A*. *loripes*, whilst their paternal species, *A*. *kenti*, is an early (at dusk) spawner (Baird et al. 2021; Harrison et al. 1984). This suggests that spawning time is maternally determined in the LK_F1_ hybrids. The fertile hybrids between *A*. *intermedia* and *A*. *florida* also spawned in synchrony with their maternal species, although the spawning times of the two parental species were very similar (Isomura et al. 2016). The lack of synchrony between the LK_F1_ hybrid and typical *A*. *kenti* spawning times could be an effective prezygotic barrier to backcrossing in this direction. It should be noted that unsynchronised spawning of the *A*. *loripes* and *A*. *kenti* colonies in nature could also be an effective prezygotic barrier to the natural production of F1 hybrids between these two species.

Although one hybrid coral spawned in 2019, no other spawning behavior was observed in the corals until 2021, when they were 6 years old. This could be due to the corals not receiving all environmental cues which enable synchronized spawning until 2021 (Fogarty and Marhaver 2019; Appendix SI). Until August 2021, the LK_F1_ and LL_F1_ corals were reared under a moonlight cycle that did not mimic natural moonrise and moonset times (Appendix SI). The length of time a coral is in darkness prior to moonrise is crucial to synchronizing spawning and thus the absence of this cue may have prevented the LK_F1_ and LL_F1_ corals from spawning at an earlier age (Lin et al. 2021; Randall et al. 2020). The fact that the F1 corals produced gametes in 2020 but were not observed to have spawned over the 10 days following the full moons between October—December 2020 is noteworthy. The corals may have spawned at a time they were not being monitored (on a day/days further from the full moon or between 23:00 and 08:00) or they may have reabsorbed the gametes without releasing them (Rinkevich and Loya 1979) either because they were not correctly cued to spawn or they were reproductively immature. While the hybrid's paternal species, *A*. *kenti*, can reach reproductive maturity at 2–4 years of age (dela Cruz and Harrison 2017; Iwao et al. 2010; Harrison et al. 2021), it is possible that its maternal species, *A*. *loripes*, becomes reproductively mature at a later age and that this trait is also maternally inherited in the hybrids; indeed, *A*. *loripes* colonies that were settled directly as larvae onto reefs in a restoration project did not reach sexual maturity within 4 years (dela Cruz and Harrison 2020). The fact that the LL_F1_ purebreds and LK_F1_ hybrids had their first mass spawning in the same year demonstrates that, under the same environmental conditions, LK_F1_ hybrids can reach reproductive maturity at the same age as LL_F1_ purebreds.

The LK_F1_ hybrids had higher reproductive fitness compared to their LL_F1_ purebred counterparts and this could be demonstrative of hybrid vigor at least for the populations studied here. The aquarium‐reared LK_F1_ hybrid and LL_F1_ populations studied here could have low genetic diversity and the relatively low reproductive fitness of the LL_F1_ population may be a product of inbreeding depression (López‐Nandam et al. 2022) with the deleterious effects of recessive alleles masked in the hybrid F1s. It is also possible that the relative fitness of the LK_F1_ and LL_F1_ corals is specific to their environment and could differ between aquarium and reef environments. In the aquarium, the hybrid colonies were more likely to spawn than the *A*. *loripes* purebreds. Furthermore, the crosses involving LL_F1_ eggs and sperm had lower fertilization success compared to the crosses that involved the LL_F1_ eggs and LK_F1_ sperm and LK_F1_ eggs × LL_F1_ sperm; the LK_F1_ eggs × LK_F1_ sperm crosses had intermediate fertilization success. However, the *A*. *loripes* purebreds crossed in a previous study to produce the LL_F1_ studied here had high fertilization success (Chan et al. 2018). This indicates the relatively low fertilization success between the LL_F1_ eggs and sperm tested here could be specific to the F1 stock. Analyses of the genomes and karyotypes of the stock would provide some indication as to the potential for interbreeding the stock beyond the F1 stages and/or any challenges due to relatedness of individuals. The mesenteries of the LK_F1_ were more likely to contain eggs than those of the LL_F1_. The LK_F1_ hybrids had larger eggs than the LL_F1_ purebreds in 2020 but neither population spawned in 2020 and the same pattern was not observed in 2021 when spawning occurred. The eggs of the corals were smaller in 2020 when they were sampled 3 weeks from the full moon than in 2021 when they were sampled 9 days from the full moon, likely because coral gametes increase in size as they mature (Wallace 1985). It is notable that several of the LL_F1_ and LK_F1_ corals were self‐fertile. Given the selfing rate within purebred acroporids is generally low (Willis et al. 1997; Heyward and Babcock 1986), it is possible that these corals were chimeras of recruits that settled in close proximity and subsequently fused (Schweinsberg et al. 2015). Unfortunately, no *A*. *kenti* purebred (KK_F1_) colonies or hybrids produced through crossing the eggs of *A*. *kenti* and sperm of *A*. *loripes* (KL_F1_) in 2015 survived to the time of this study in the aquarium systems, hence the reproductive capacity of the LK_F1_ hybrids could not be compared to that of *A*. *kenti* or the reciprocal hybrid (KL_F1_).

While we demonstrated compatibility between LK_F1_ hybrids and purebred *A*. *loripes* (in the maternal direction), crosses between LK_F1_ and *A*. *kenti* are required to discern the ability of the hybrid to backcross in the paternal direction. Bidirectional introgression has been detected between *A*. *palmata* and *A cervicornis* and indicates that the hybrid between the two species, *A*. *prolifera*, is capable of backcrossing with both parental purebred species (Kitchen et al. 2020; Japaud et al. 2019). Hybrids of *A*. *intermedia* and *A*. *florida* have also been successfully crossed with colonies of both of their parental species (Isomura et al. 2016).

Outbreeding depression is a reduction in the fitness of first or later generations of hybrids between populations of species or species that occurs due to genetic incompatibilities (Frankham et al. 2011). The risk of outbreeding depression increases when the crossed populations belong to different species (Frankham et al. 2011) and can increase at the F2 stage because errors in recombination within the gametes of the F1 can result in an F2 with genomic aberrations such as chromosome losses or gains (Benirschke, Brownhill, and Beath 1962; White 1977). *Acropora kenti*, like most tested acroporids, has 28 somatic chromosomes (Kenyon 1997), while the chromosome number of *A*. *loripes* is unknown. Comparisons of the karyotypes and chromosome‐level whole genome sequences of *A*. *kenti* and *A*. *loripes* may help in making predictions regarding the likelihood of outbreeding depression occurring in their hybrids. However, *A*. *loripes* and *A*. *kenti* have now been successfully crossed to produce an F1 generation that is capable of surviving, growing, and reaching reproductive maturity (Chan et al. 2018; Chan, Peplow, and van Oppen 2019). Moreover, the F2 corals produced in this study successfully progressed through their early life stages: the fertilized embryos developed to planula larvae and the planula coral larvae settled and metamorphosed into their sessile polyp form. Nevertheless, although the F1 and F2 generations of the *A*. *loripes* and *A*. *kenti* hybrid lineage appear to be viable, it is possible that outbreeding depression may be expressed in a later F2 life stage (e.g., adults), in later generations, in a different environment (particularly on the reef), or for different traits not measured here (e.g., size and growth rate of recruits).

### 3.2 Interspecific Hybridization as a Potential Reef Restoration Tool

Interspecific hybridization has been proposed as a tool for the management of coral reefs (Chan, Hoffmann, and van Oppen 2019). Several studies have shown that coral hybrids can have higher fitness for multiple traits compared to one or both of their purebred counterparts and therefore might constitute resilient stock for reef restoration initiatives (VanWynen et al. 2021; Fogarty 2012; Chan et al. 2018; Willis et al. 1997). The results obtained here indicate that coral hybrids may contribute to reef restoration beyond an outplanted F1 generation, adding potentially resilient biomass to reefs. Firstly, fertile hybrids are capable of intercrossing and/or backcrossing to produce novel genetic combinations, adding to the genetic diversity of the region, and potentially to the adaptive potential of receiving populations. This can be particularly important in the case of degraded systems with low effective population sizes and decreased genetic variation. Hybrids might therefore serve to genetically rescue certain populations. Fertile hybrids will also act to perpetuate the genomic information of their parental species, which is particularly important in cases where those species are vulnerable to climate and other stressors and face extinction. An opposing perspective is that hybridization can reduce species‐level diversity by facilitating genetic mixing that reduces genetic distinctiveness amongst species (Allendorf et al. 2001). Active management programs that consider incorporating hybridization‐based interventions must balance the risks and benefits of maintaining genetic uniqueness at the species level against those of maximizing adaptive capacity. The virtue of using hybridization to rescue endangered species has been demonstrated by the inter‐subspecific crosses that have rescued the Florida panther (Land and Lacy 2000; Hedrick 1995), South Island robin (Heber et al. 2012), and Norfolk Island boobook owl (Garnett et al. 2011).

## 4 Materials and Methods

### 4.1 Coral Stock Generation and Aquarium Rearing

Following the full moon on the 22/11/2015, during a GBR coral mass spawning period, hybrid and purebred corals were generated to test the performance of interspecific coral hybrids as described in Chan, et al. (2018). Briefly, gravid *A*. *loripes* and *A*. *kenti* (previously referred to as *A*. *tenuis*; Bridge et al. 2023) colonies were collected from Trunk Reef (−18.302 S, 146.869 E) and brought to the SeaSim at AIMS prior to the full moon. Acroporids are broadcast spawners that participate in coral mass spawning events when colonies of these species release buoyant packages of their gametes (egg‐sperm bundles) into the water column which cross‐fertilize to produce offspring (Harrison et al. 1984). *Acropora kenti* is an early (at dusk) spawner whilst *A*. *loripes* typically spawns later in the evening (Baird et al. 2021; Harrison et al. 1984). Two purebred and two hybrid offspring groups were created from the gametes of these two species through in vitro hybridization in the initial study by Chan, et al. (2018): the eggs of six *A*. *loripes* dams and sperm of five *A*. *kenti* sires were crossed to produce the LK_F1_ hybrid stock, the eggs and sperm of six *A*. *loripes* colonies were crossed to produce the LL_F1_ purebred stock, the eggs of five *A*. *kenti* dams and sperm of five *A*. *loripes* sires were crossed to produce the KL_F1_ hybrid offspring, and the eggs and sperm of five *A*. *kenti* colonies were crossed to produce the KK_F1_ purebred stock. It should be noted that multiple recruits from a given F1 group could settle onto individual plugs such that the colonies studied herein could be chimeras.

The hybrid and purebred coral were randomized amongst 24 replicate tanks in a 28‐week experiment under either ambient (27°C, 415 ppm) or elevated (28°C, 685 ppm) temperature and *p*CO_2_ conditions (Chan et al. 2018). After the 28 weeks, surviving corals were randomized amongst holding systems in the SeaSim. The details of the coral rearing conditions are outlined in Appendix SI. As of December 2021, 31 LL_F1_ (~10% post‐settlement survival) and 39 LK_F1_ corals (~7% post‐settlement survival) had survived, whilst there were no surviving KK_F1_ or KL_F1_ corals. The surviving LL_F1_ and LK_F1_ became the F1 parental groups of this experiment. Over the first 28 weeks of their lives, 19 of the LL_F1_ and 24 of the LK_F1_ colonies were exposed to ambient conditions and 12 of each of the LL_F1_ and LK_F1_ colonies were exposed to elevated conditions; the treatment that three of the LK_F1_ were exposed to was not tracked.

### 4.2 F1 Gametogenesis

Fragments of the LL_F1_ purebred and LK_F1_ hybrid F1 parental corals were dissected to examine the presence of maturing oocytes. Three weeks prior to the 30/11/2020 full moon, 13 LL_F1_ and 16 LK_F1_ corals were sampled, and 9 days prior to the 19/12/2021 full moon, 16 LL_F1_ and 16 LK_F1_ corals were sampled from across the four tanks of each of the two holding systems; note that some of the same (5 LL_F1_ and 6 LK_F1_) and some different colonies were sampled between the two time points. One fragment with a length of 13–46 mm that contained greater than 20 polyps was sampled from each colony and fixed for several days in 10% formaldehyde in filtered seawater (0.2 μm). Formalin is commonly used to fix eggs of scleractinian corals (Wallace 1985) and does not affect the validity of findings pertaining to egg size in other spawning species (Nyuji et al. 2022). Following fixation, the coral skeleton was dissolved using a 3% solution of hydrochloric acid in purified reverse osmosis (RO) water solution. The acid solution was replaced every 1–3 days until the skeleton was completely dissolved. The decalcified samples were stored in RO water for several months and then in 10% formaldehyde for long‐term storage until they were dissected.

Dissections took place under a dissecting microscope and were imaged using the ToupCam Industrial Digital Camera C‐Mount Microscope Eyepiece and ToupLite imaging software. ToupLite was calibrated using a micrometer calibration and the horizontal line tool. Each fragment was examined for the presence of a sterile zone—a non‐gravid region that has been observed in coral species and is typically associated with new growth at the tips of branching acroporids (Randall, Giuliano, and Page 2021; Wallace 1985). Ten polyps were selected at random from each sample and the mesenteries of those polyps were spread out. Acroporid coral polyps contain eight reproductive mesenteries, with four containing oocytes and four containing spermaries (Wallace 1985). The number of maturing oocytes in the four mesenteries of each polyp were counted. All statistical analyses in this experiment were conducted in R Core Team (2021). The number of oocytes in the mesenteries of the LL_F1_ and LK_F1_ corals were compared using a zero‐inflated generalized linear mixed effects model that tested the effect of parental group (LL_F1_ or LK_F1_), year of sampling (2020 or 2021), and an interaction between parental group and year of sampling on the number of oocytes per mesentery using a Poisson link function (Brooks et al. 2017). The variation due to the nested random effects of mesenteries within polyps, polyps within colonies and colonies within holding systems, and the random effects of the temperature treatment the colonies experienced over the first 28 weeks of their lives was accounted for. The lsmeans package (Lenth 2016) was used to conduct a post hoc Tukey's test to compare the number of eggs per mesentery between parental groups for each year.

The size of the oocytes in the mesenteries were also compared between the LL_F1_ and LK_F1_ corals. Each of the 10 dissected polyps was imaged at 2.5 × magnification. If one of the 10 dissected polyps did not contain oocytes, an additional polyp with oocytes was dissected, unless no gravid polyps remained in the sample. The ToupLite line tool was utilized to measure the size of each oocyte in millimeters in four randomly‐selected polyps from each sample. Note that some samples contained fewer than four gravid polyps, in which case all gravid polyps were analyzed. Two perpendicular lengths of each oocyte in the polyps were measured and the mean of these values was taken to get an average diameter for each oocyte. An LMM was constructed to test whether average oocyte diameter differed between the sampling time points and the parental groups using the lmer function in the lme4 package (Bates et al. 2014). The variation due to the nested random effects of eggs within mesenteries, mesenteries within polyps, polyps within colonies and colonies within holding systems, and the random effects of the temperature treatment the colonies experienced over the first 28 weeks of their lives was accounted for. A post hoc Tukey's test was used to compare the average oocyte diameter between years and between parental groups for each year.

### 4.3 F1 Spawning Activity

In 2019, 2020, and 2021, the F1 parental corals were observed nightly for 7–10 days following the October, November, and December full moons for spawning activity during the predicted spawning periods. To prevent uncontrolled cross‐fertilization of their gametes, the colonies were isolated during the sunset period in individual plastic bags that sat inside the holding systems and where the upper rim of each bag rose above the water level. The colonies did not bleach or slough their tissue during or after their spawning season, indicating they were not stressed by the method of isolation. One LK_F1_ coral spawned in 2019, no spawning was observed in 2020, and a total of 21 of the surviving 31 LL_F1_ and 32 of the surviving 39 LK_F1_ corals spawned in 2021.

Minutes to hours prior to spawning, acroporid polyps generally indicate their imminent spawning by their egg‐sperm bundles bulging under their mouths such that the colony appears ‘set’ (sensu Harrison et al. 1984). The date and time that each coral began spawning was recorded. To compare the spawning output of the LK_F1_ and LL_F1_ corals in 2021, a GLMM was built using the glmer function from the package lme4 (Bates et al. 2014). In the model, whether the colony spawned or not on a given night was considered a binary response variable and a binomial link function was applied. The random variation amongst spawning dates was accounted for, as was the random variation due to the nested effects of colonies within holding systems and the temperature treatment the colonies experienced over the first 28 weeks of their lives. The spawning times of the two parental groups were further compared using an LMM and the lme function from the nlme package (Pinheiro et al. 2021). The LMM tested the effect of parental group on the number of minutes after sunset (18:50) that the coral spawned, whilst accounting for the random variation due to the nested effects of colonies within holding systems.

### 4.4 F2 Fertilization Success

To test the ability of the gametes of the LK_F1_ and LL_F1_ corals to produce offspring, fertilization tests were conducted. Over the 2021 December spawning event, the egg‐sperm bundles of F1 colonies were collected, and the eggs were separated from the sperm of the bundles by gently agitating them in a container for several minutes with a 100 μm filter mesh at the base, in 1 μm filtered sea water (FSW). The eggs were washed three times in FSW to ensure they were sperm‐free. Controlled crosses of the gametes were conducted where 100 eggs were combined with sperm at a density of ~1 × 10^6^ sperm per mL in 10 mL reactions in six‐well plates of FSW ~ 1–2.5 h post‐spawning. Each F1 colony involved in the crosses was assessed for its ability to self‐fertilize by combining its eggs and sperm in duplicate reactions. Samples of 100 eggs from each colony were also taken and not combined with any sperm to test for contamination of the egg samples with compatible sperm.

In December 2021, gravid *A*. *loripes* and *A*. *kenti* colonies were not available for crossing (colonies collected for this purpose spawned in November 2021), hence the LL_F1_ and LK_F1_ corals could only be crossed with one another. The following crosses were conducted in duplicate reactions: 18 crosses between unique combinations of the eggs and sperm of different LL_F1_ purebreds to produce LLLL_F2_ corals, 18 crosses between unique combinations of the eggs of a LL_F1_ purebred and the sperm of a LK_F1_ hybrid to produce LLLK_F2_ corals, 18 crosses between unique combinations of the eggs of a LK_F1_ hybrid and the sperm of a LL_F1_ purebred to produce LKLL_F2_ corals, and 28 crosses between unique combinations of the eggs and sperm of different LK_F1_ hybrids to produce LKLK_F2_ corals (Table 1). Fertilization success was counted between 1.75 and 3.5 h post‐mixing of the eggs and sperm. The effect of offspring group (Table 1) on fertilization success (number of fertilized eggs) was assessed using a BGLMM, the brms package in R, and a Poisson link function (Bürkner 2017). The random variation in the performance of individual colonies as dams and sires and the random variation between duplicate reactions were accounted for. A post hoc analysis of the model output analyzed the estimated marginal means using the contrast function in the package emmeans (Lenth 2022) to compare the fertilization success amongst the offspring groups in a pairwise manner.

### 4.5 F2 Viability

Embryos from successful crosses conducted in 2021 were transferred to 12‐L conical tanks for rearing through to their planula larval stage. Once competent to settle, the larvae were added to 50 L acrylic tanks containing ceramic plugs that had been biologically conditioned for approximately 2 months in the rearing tanks of the F1 corals to promote ‘settlement’. Larvae initially attached to the plugs and then metamorphosed into sessile coral recruits during settlement. Upon settlement, the coral larvae were exposed to Symbiodiniaceae that had been isolated from the tissue of their parents. To produce a symbiont slurry, soft tissue was removed from ~5 cm long parental fragments using an airbrush into FSW. Symbiodiniaceae were isolated and washed by three rounds of centrifuging the extract at 2000 *g* for 5 min and resuspending the pellet in FSW to produce a solution that was added to the settlement tanks at a density of ~2 × 10^6^ cells per mL. Settled recruits were reared for several months in 50‐L acrylic tanks and then transferred to the holding systems of their parents until the time of this publication. Different numbers of LLLL_F2_, LLLK_F2_, LKLL_F2_, and LKLK_F2_ corals were produced, and the different offspring groups were reared in separate 50‐L acrylic tanks, such that the relative settlement success and survivorship of the groups could not be assessed. However, the progression of the F2 offspring was monitored to assess their viability over their early life stages.

## Author Contributions

**Annika M. Lamb:** conceptualization (lead), data curation (lead), formal analysis (lead), funding acquisition (supporting), investigation (lead), methodology (lead), project administration (equal), resources (equal), validation (lead), visualization (lead), writing – original draft (lead), writing – review and editing (lead). **Lesa M. Peplow:** conceptualization (supporting), data curation (supporting), investigation (supporting), methodology (supporting), project administration (supporting), resources (supporting), supervision (supporting), validation (supporting), writing – original draft (supporting), writing – review and editing (supporting). **Wing Yan Chan:** conceptualization (supporting), data curation (supporting), investigation (supporting), project administration (supporting), resources (supporting), writing – original draft (supporting), writing – review and editing (supporting). **Zoe J. Crane:** data curation (supporting), formal analysis (supporting), investigation (supporting), methodology (supporting), resources (supporting), visualization (supporting), writing – original draft (supporting), writing – review and editing (supporting). **Glenn A. Everson:** investigation (supporting), methodology (supporting), project administration (supporting), resources (supporting), writing – original draft (supporting), writing – review and editing (supporting). **Peter L. Harrison:** conceptualization (supporting), investigation (supporting), methodology (supporting), project administration (supporting), supervision (supporting), writing – original draft (supporting), writing – review and editing (supporting). **Talley E. Hite:** data curation (supporting), formal analysis (supporting), investigation (supporting), methodology (supporting), resources (supporting), writing – original draft (supporting), writing – review and editing (supporting). **Ary A. Hoffmann:** conceptualization (supporting), formal analysis (supporting), investigation (supporting), project administration (supporting), resources (supporting), supervision (supporting), writing – original draft (supporting), writing – review and editing (supporting). **Craig A. Humphrey:** conceptualization (supporting), funding acquisition (supporting), investigation (supporting), project administration (supporting), resources (supporting), supervision (supporting), writing – original draft (supporting), writing – review and editing (supporting). **Lonidas P. Koukoumaftsis:** investigation (supporting), methodology (supporting), resources (supporting), validation (supporting), writing – original draft (supporting), writing – review and editing (supporting). **Madeleine J. H. van Oppen:** conceptualization (lead), data curation (supporting), formal analysis (supporting), funding acquisition (lead), investigation (supporting), methodology (supporting), project administration (lead), resources (lead), supervision (lead), validation (supporting), visualization (supporting), writing – original draft (supporting), writing – review and editing (supporting).

## Conflicts of Interest

The authors declare no conflicts of interest.

## Supporting information


Data S1.

## Data Availability

Data and code are publicly available on a GitHub data repository: https://github.com/AnnikaMLamb/Fertile‐hybrids‐could‐aid‐coral‐adaptation.
