# Supplementary material for: Fertile Hybrids Could Aid Coral Adaptation
Source: Ecol Evol. 2024 Nov 20;14(11):e70570. doi: 10.1002/ece3.70570 (PMC11578633; doi:10.1002/ece3.70570)
Supplement: Supplementary file 1 — Data S1. [file ECE3-14-e70570-s001.docx]

**Supporting Information for**

Fertile hybrids could aid coral adaptation.

Authors: Annika M. Lamb^1,2,3^*, Lesa M. Peplow^1^, Wing Yan Chan^1,2^, Zoe J. Crane^1^, Glenn A. Everson^1^, Peter L. Harrison^4^, Talley E. Hite^1^, Ary A. Hoffmann^25^, Craig A. Humphrey^1^, Lonidas P. Koukoumaftsis^1^, and Madeleine J.H. van Oppen^1,2^

^1^Australian Institute of Marine Science - 1526 Cape Cleveland Road, Cape Cleveland 4810, Queensland, Australia

^2^School of Biosciences, University of Melbourne - Biosciences 4, The University of Melbourne, Royal Parade, Parkville VIC 3052

^3^AIMS@JCU - James Cook University, Townsville, QLD 4811, Australia

^4^Marine Ecology Research Centre - Southern Cross University, Lismore NSW 2480

^5^School of Biosciences, Bio21 Institute - University of Melbourne, Melbourne VIC 3010

*Annika M. Lamb

Email: a.lamb@aims.gov.au

Supporting Information Text

**Coral rearing conditions**

The F1 corals studied here were used in a 28-week experiment under either ambient (27°C, 415 ppm) or elevated (28°C, 685 ppm) temperature and *p*CO_2_ conditions (1). After 28 weeks of experimentation, surviving corals were housed in a holding system, comprised of three 250 L tanks at a constant temperature of 27.5°C in the SeaSim. The corals were held under a maximum of 225 PAR under artificial lighting (Aqua Illumination® Hydra®) running on a daylight profile simulating a 7:00 am sunrise and a 16:30 sunset, with a two-hour ramp-up and ramp-down period on either end of the day to mimic sunrise and sunset, respectively. In April 2019, the corals were spread amongst four tanks in each of two holding systems to provide them with room for growth; one holding system comprised of four 280 L tanks (three of these contained the corals prior to this move) and the other comprised of 280 L tanks and two 1200 L tanks. Annual temperature cycling and lunar phases were simulated in the holding systems because these environmental variables cue the mass spawning of corals on reefs (2). Temperature cycling began in the holding systems according to the 18-year (1998 – 2015) daily averages recorded (3) at six meter depth at Davies Reef (-18.828 S, 147.643 E; ~ 100 km south east of Trunk Reef) on the 16/05/2019. From the 21/05/2020, the daylight profile was changed weekly to match the daylight length at Townsville (4). On the 20/06/2019, additional Hydra lights were installed and profiled to emit 1 lx of blue light on the date of the full moon in Townsville (the value measured under a full moon at AIMS) and a percentage of 1 lx relative to the moon phase for a constant time between 21:00 pm and 5:00 am (4). From the 30/08/2021, the moonrise and moonset times in the holding systems were changed daily to match those of the actual moon in Townsville and the percentage of 1 lx emitted by the lights was altered according to the moon illumination percentage listed on timeanddate.com (4).

**2019 spawning observations**

On the 18/12/2019 (six days following the full moon on the 12/12/2019), one LK_F1_ coral spawned and constituted the first spawning observation in the LL_F1_ and LK_F1_ corals. In December 2019, gravid *A. loripes* and *A. kenti* colonies were available at AIMS that had been collected from Davies Reef (~100 km south-east of Trunk Reef, the reef from which the parents of the F1 LL_F1_ and LK_F1_ corals were collected in 2015; GBRMPA Permit G12-35236.1) for backcrossing with the captive-reared F1 corals. The LK_F1_ coral began spawning (85 minutes after sunset) between the times that six colonies of the maternal species, *A. loripes* (175 – 189 minutes after sunset), and five colonies of the paternal species, *A. kenti* (42 – 53 minutes after sunset), began spawning on the same night.

The bundles of the colonies were collected, and the eggs were separated from the sperm of the bundles by gently agitating them in a container with a 100 μm filter mesh at the base, in 1 μm filtered sea water (FSW). The eggs were washed three times in FSW to ensure they were sperm-free. Controlled crosses of the gametes were conducted where 100 eggs were combined with sperm at a density of ~ 1 X 10^6^ sperm per mL in 10 mL reactions in six-well plates ~ 1 – 2.5 hrs post-spawning. Each parental colony involved in the crosses was assessed for its ability to self-fertilize by combining its eggs and sperm in duplicate reactions. Samples of 100 eggs from each colony were also taken and not combined with any sperm to test for contamination of the egg samples with compatible sperm.

The gametes of the LK_F1_ hybrid that spawned in 2019 were crossed with those of two *A. kenti* and two *A. loripes* colonies collected from Davies Reef to assess its ability to backcross (Table S1). The number of crosses that could be conducted were limited by the small number of gametes released by the LK_F1_ hybrid (approximately 30-40 bundles) and each cross was done in duplicate reactions. The sperm of the LK_F1_ hybrid were crossed with the eggs of each of the purebred colonies in separate reactions while the eggs of the LK_F1_ hybrid were crossed with the sperm from the pair of conspecific purebreds in the same reaction. The gametes of the conspecific pairs of field-collected corals were crossed to ensure their viability (Table S1). The number of fertilized eggs after 2 – 2.5 hrs in each reaction was counted as a measure of fertilization success.

The eggs and sperm of the one LK_F1_ hybrid colony that spawned in 2019 were capable of backcrossing with gametes of its maternal species, *A. loripes*, but incompatible with the gametes of its paternal species, *A. kenti*. The eggs of the LK_F1_ colony were fertilized by the sperm of the two *A. loripes* colonies tested (98 – 99% egg fertilization) but not by the sperm of the two *A. kenti* colonies (0%). Similarly, the sperm of the LK_F1_ hybrid successfully fertilized the eggs of the two *A. loripes* colonies (79 – 96%) but not the eggs of the *A. kenti* colonies (0%). The pair of *A. loripes* colonies were not compatible with each other (0%), while the pair of *A. kenti* colonies were compatible (90 – 95%), based on fertilization success of the crosses between them.

Embryos from successful crosses conducted in 2019 were transferred to 12 L conical tanks for rearing through to their planula larval stage, after which they were added to 50 L acrylic tanks containing ceramic plugs that had been biologically conditioned in coral rearing tanks to promote ‘settlement’. Larvae settled, attached to the plugs, and metamorphosed into sessile coral recruits. Upon settlement, the coral larvae were exposed to Symbiodiniaceae that had been isolated from the tissue of their parents. To produce a symbiont slurry, soft tissue was removed from parental fragments using an airbrush into FSW. Symbiodiniaceae were isolated and washed by conducting three rounds of centrifuging the extract at 2000 *g* for five minutes and resuspending the pellet in FSW to produce a solution that was added to the settlement tanks. Settled recruits were reared for the short term in 50 L acrylic tanks and in the holding systems of their parents for the longer term.

Table S1. Offspring groups resulting from the crosses of the eggs and sperm of the various parental coral groups conducted in the 2019 December spawning season.

| **Dam** | **Sire** | **Offspring Group** |
| --- | --- | --- |
| LK_F1_ | *A. loripes* from Davies Reef | LKLL_F2_ |
| LK_F1_ | *A. kenti* from Davies Reef | LKKK_F2_ |
| *A. loripes* from Davies Reef | LK_F1_ | LLLK_F2_ |
| *A. kenti* from Davies Reef | LK_F1_ | KKLK_F2_ |
| *A. loripes* from Davies Reef | *A. loripes* from Davies Reef | LLLL_F1_ |
| *A. kenti* from Davies Reef | *A. kenti* from Davies Reef | KKKK_F1_ |

**SI cited literature**

1. W. Y. Chan, L. M. Peplow, P. Menéndez, A. A. Hoffmann, M. J. H. van Oppen, Interspecific Hybridization May Provide Novel Opportunities for Coral Reef Restoration. *Frontiers in Marine Science* **5** (2018).

2. P. L. Harrison *et al.*, Mass spawning in tropical reef corals. *Science* **223**, 1186-1189 (1984).

3. Australian Institute of Marine Science (2017) AIMS Sea Water Temperature Observing System (AIMS Temperature Logger Program).

4. Time and Date AS (2022) timeanddate.com.
